# Racially Disparate Expression of mTOR/ERK-1/2 Allied Proteins in Cancer

**DOI:** 10.3389/fcell.2021.601929

**Published:** 2021-04-30

**Authors:** Sanjay Mishra, Manish Charan, Ajeet Kumar Verma, Bhuvaneswari Ramaswamy, Dinesh Kumar Ahirwar, Ramesh K. Ganju

**Affiliations:** ^1^Department of Pathology, Wexner Medical Center, College of Medicine, The Ohio State University, Columbus, OH, United States; ^2^Comprehensive Cancer Center, The Ohio State University, Columbus, OH, United States

**Keywords:** racial disparity, cancer, mTOR, ERK, MAPK

## Abstract

Recent studies revealed that ethnic differences in mechanistic target of rapamycin (mTOR) and extracellular signal-regulated kinase (ERK-1/2) signaling pathways might be associated with the development and progression of different human malignancies. The African American (AA) population has an increased rate of cancer incidence and mortality compared to the Caucasian American (CA) population. Although the socioeconomic differences across different ethnic groups contribute to the disparity in developing different cancers, recent scientific evidence indicates the association of molecular and genetic variations in racial disparities of different human malignancies. The mTOR and ERK-1/2 signaling pathways are one of the well-known oncogenic signaling mechanisms that regulate diverse molecular and phenotypic aspects of normal as well as cancer cells in response to different external or internal stimuli. To date, very few studies have been carried out to explore the significance of racial disparity with abnormal mTOR and ERK-1/2 kinase signaling pathways, which may contribute to the development of aggressive human cancers. In this review, we discuss the differential regulation of mTOR and ERK-1/2 kinase signaling pathways across different ethnic groups, especially between AA and CA populations. Notably, we observed that key signaling proteins associated with mTOR and ERK-1/2 pathway including transforming growth factor-beta (TGF-β), Akt, and VEGFR showed racially disparate expression in cancer patients. Overall, this review article encompasses the significance of racially disparate signaling molecules related to mTOR/ERK1/2 and their potential in developing tailor-made anti-cancer therapies.

## Introduction

Recent reports on cancer research highlighted the significant impact of racial disparities on the clinical outcome of human malignancies (Esnaola and Ford, 2012; Heath et al., 2018; Freedman et al., 2021; Zavala et al., 2021). Cancer is one of the leading causes of death worldwide and witnessed major advancements in terms of cancer prevention, management, and newer treatments over the past few decades. Although these advancements have indeed improved the quality of life of cancer patients, statistical data on cancer-related mortalities, pointed out an inconsistency among certain ethnic groups regarding the disease incidence, aggressiveness, and clinical outcome (Gross et al., 2008; Esnaola and Ford, 2012). Cancer-related racial disparities have been reported among all ethnicities but the racial disparity has been extensively analyzed between the Caucasian American (CA) and African American (AA) population.

Many clinical factors such as aberrant genomic mutations, immune cell response, epigenetic modifications, and deregulated cell signaling pathways show racially disparate expression in several human cancers (Charan et al., 2020b). In recent years, numerous research studies around the world, have established a crucial role of the mechanistic target of rapamycin (mTOR) pathway in cellular growth and survival of mammalian cells (Soliman, 2013). mTOR is a Ser/Thr protein kinase that plays a key role in regulating cellular metabolism and immunity (Soliman, 2013). The dysregulation of mTOR pathway often leads to the initiation and progression of cancer. Several novel functions of the mTOR pathway in cancers have been discovered lately. mTOR is another essential kinase whose targeting has shown improved clinical efficacy against renal carcinoma (Battelli and Cho, 2011). Racial differences in African American renal cell carcinoma patients are well-recognized (Krishnan et al., 2016). Temsirolimus and everolimus are two FDA-approved drugs that exert anti-tumor effects through mTOR pathway inhibition (Voss et al., 2011). In a phase I study, everolimus showed higher renal clearance in AA population and, therefore, higher doses were required to achieve equivalent effects as observed in non-black patients (Kovarik et al., 2001).

The mitogen-activated protein kinase (MAPK) signaling is one of the most extensively investigated pathways that constitute Ras, Raf, mitogen-activated protein/extracellular signal-regulated kinase (MEK), and extracellular signal-regulated kinase (ERK-1/2; Morrison, 2012). MAPK signaling cascade is highly evolutionarily conserved signaling and partakes in a myriad of cellular functions including cell survival, proliferation, growth, and apoptosis (Braicu et al., 2019). MAPK pathway acts as a connecting link between extracellular signals and intracellular responses. Atypical genetic and epigenetic changes that lead to dysregulation of this pathway often result in the development of cancer (Braicu et al., 2019). Altered expression of MAPK family kinases has been reported in various cancers and many targeted therapies against these kinases have been tested in multiple human malignancies (Lee et al., 2020). Recently, a research study indicated MAPK/MEK1 mutations in AA colorectal cancer patients (Heath et al., 2018). Although there is no direct evidence of the racially disparate expression of the ERK-1/2 pathway in cancers, ERK-1/2 might be activated indirectly *via* different signaling cascades including MAPK/MEK pathway in AA patients. However, the development of resistance and redundancy in mTOR and ERK-1/2 pathways still present a major hurdle for precision medicine.

The interplay between mTOR and ERK-1/2 signaling pathways is a key determinant of cancer progression and metastasis (Mendoza et al., 2011). However, the exact underlying molecular mechanism connecting mTOR and ERK-1/2 signaling is not yet established. In this review article, we have summarized the current understanding of the mTOR pathway and its varied signaling networks. Until date, not much is known about the racially disparate expression of mTOR and ERK-1/2 pathways in cancers. We have comprehensively reviewed cancer-related racial disparities and the expression or activation status of the upstream regulator and downstream effector proteins that regulate the mTOR and ERK-1/2 pathway.

Here, we focus on current understanding of mTOR and MAPK pathways in cancer, with a particular emphasis on racially disparate regulation of these signaling pathways and their crosstalk with other key signaling molecules in different solid tumors. We highlight the mTOR pathway in response to intra- and extracellular signals that regulate energy homeostasis, cell growth, proliferation, and survival. Although mTOR targeting is clinically approved for treatment of renal cancers, overall survival for AA renal cancer patients compared to CA counterpart has not been improved. We will review the current approaches of targeting mTOR and ERK-1/2 signaling using pharmacological inhibitors and their therapeutic relevance in AA cancer patients.

## mTOR/ERK-1/2 Signaling

Rapamycin was first extracted from the soil of Easter Island during an expedition. Later, the Ayerst Pharmaceuticals identified an antifungal macrolide from this soil, which was produced from *Streptomyces hygroscopicus* bacterium (Seto, 2012). This compound was named Rapamycin attributed to its place of origin, Rapa Nui (Easter Island). Further characterization of Rapamycin revealed its potential as an immunosuppressive and anti-tumor agent (Xie et al., 2016). However, its mechanism of action remained obscure for almost two decades until 1994 when biochemical analysis characterized the mTOR as the direct target of the rapamycin-FKBP12 complex in mammals and established a critical role of mTOR in cell signaling nexus (Kennedy and Lamming, 2016). mTOR plays a central role in regulating a variety of fundamental cell processes, including cell survival, proliferation, protein synthesis, and autophagy (Kim and Guan, 2019).

**Figure 1:**
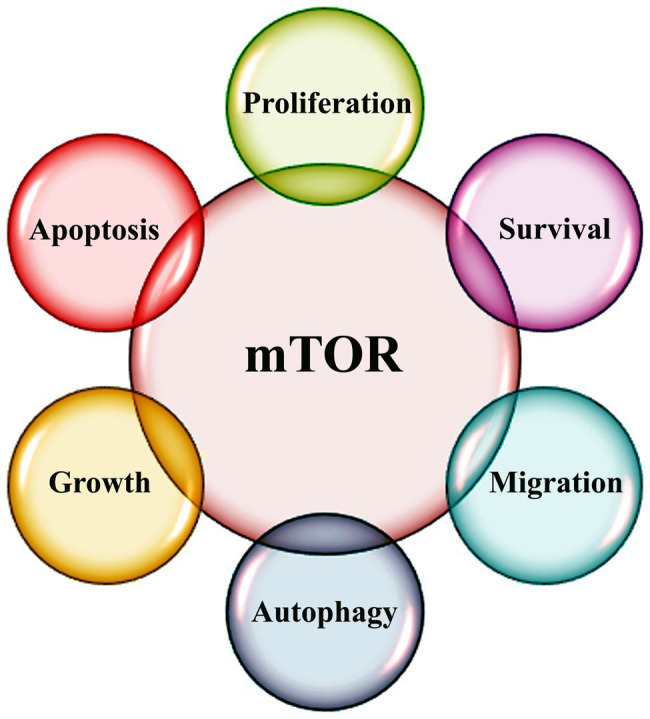
Schematic diagram highlighting various functions of mTOR signaling.

Genetic and metabolic changes influence hallmark characteristics of cancer that are indispensable for tumor initiation, growth, and metastasis. mTOR is a key determinant of cellular metabolism and signaling pathways (Figure 1). Cellular defects in energy homeostasis cause the activation of phosphatidylinositol 3-kinase (PI3K)/AKT/mTOR pathway (Fruman et al., 2017). mTOR is a Ser/Thr protein kinase and belongs to the PI3K-related kinase family and forms two distinct multi-protein complexes, mTOR complex 1 (mTORC1) and 2 (mTORC2; Zhou and Huang, 2011). mTORC1 consists of mTOR, mLST8, and Raptor is the most extensively characterized mTOR component (Saxton and Sabatini, 2017). In presence of specific growth factors, mTORC1 is activated and regulates cellular growth and proliferation through eukaryotic translation initiation factor 4E (eIF4E), ribosomal protein S6 kinases, and eIF4E-binding proteins (eIF4E-BP1, 2, and 3; Josse et al., 2016).

Besides, p53 family members have been reported to regulate mTOR signaling (Rosenbluth et al., 2008; Berkers et al., 2013). p53 and its family members; p63 and p73 are well-known tumor suppressor proteins that play a crucial role in preventing the oncogenic transformation of cells (Charan et al., 2020a). Also, p53, p63, and p73 ensure normal cellular fate by mediating a critical role in sensing different cellular cues and coordinating their downstream responses including cell senescence, cell-cycle arrest, differentiation, and cell death (Collavin et al., 2010; Cam et al., 2020). Moreover, an integrated network of p53 family members and mTOR pathway ensures homeostasis in a normal cell. However, the exact molecular mechanism of p53 family members mediated mTOR regulation is still not clear. mTOR pathway is often deregulated in different cancers (Kim et al., 2017). Dysregulation of the mTOR pathway disturbs the homeostasis of cells that may help in the metastasis of cancers.

**Figure 2:**
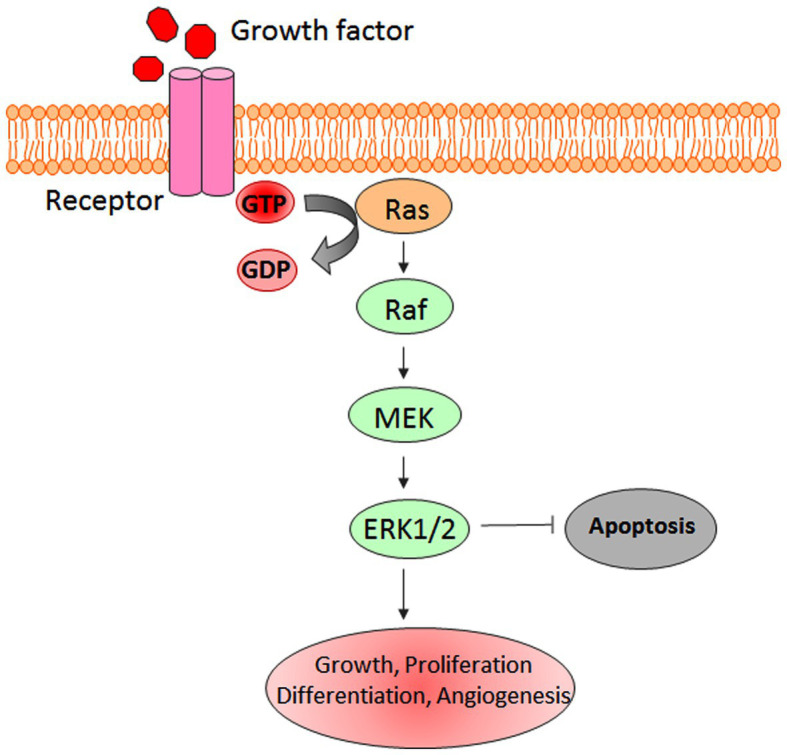
Schematic representation of extracellular signal-regulated kinase (ERK-1/2) pathway.

Mitogen-activated protein kinases regulate key cellular processes and signaling pathways, including cell growth, survival, differentiation, and programmed cell death. To date, six different types of MAPKs have been identified in mammalian systems, which include extracellular signal-regulated kinase (ERK)1/2, ERK3/4, ERK5, ERK7/8, Jun N-terminal kinase (JNK)1/2/3, and the p38 isoforms α/β/Ɣ(ERK6)/ẟ (Schaeffer and Weber, 1999; Krens et al., 2006; Dhillon et al., 2007; Kyriakis and Avruch, 2012). ERK-1/2 was the first discovered MAPK that plays the main role in survival and proliferation signaling pathways (Wortzel and Seger, 2011). MAPK signaling pathways play a key role in regulating the responses to various extra and intracellular stress stimuli (Figure 2; Munshi and Ramesh, 2013).

Anomalous activation of the ERK-1/2 pathway has been associated with tumorigenesis (Deschenes-Simard et al., 2014). Hyperactivation of ERK-1/2 signaling has been reported in several malignancies including neuroblastoma, renal, ovarian, breast, lung, and colorectal cancers (Steinmetz et al., 2004; Vicent et al., 2004; Bartholomeusz et al., 2012; Roseweir et al., 2018; Xie et al., 2019; Zhu et al., 2020). ERK-1/2 signaling cascade also promotes the invasive and metastatic abilities of cancer cells (Ward et al., 2001). Besides, ERK-1/2 plays a key role in enabling cancer cells with development of chemo- and radio-resistance and regulates the tumorigenic potential through the maintenance of cancer stem cells (Ciccarelli et al., 2016; Salaroglio et al., 2019).

## mTOR/ERK-1/2-Associated Signaling Molecules and Racial Disparity

Although mTOR and ERK-1/2 signaling pathways have been well-studied in several human cancers, their association with racial disparity has never been explored. AA population tends to have a higher number of gene mutations compared to the CA cohort (Chang et al., 2019). Genomic analyses revealed that AA breast cancer patients show 28,847 single-nucleotide polymorphisms (SNPs) in intronic regions of 61 genes that were associated with the mTOR pathway (Cheng et al., 2016). However, not much has been investigated on mTOR/ERK-1/2 genomic alterations in different human malignancies. Although a large number of signaling molecules are associated with mTOR/ERK-1/2 signaling pathways, very few studies have reported their differential expression in different races of cancer patients.

Recent reports have highlighted the disparate expression of transforming growth factor-beta (TGFβ) in different pathophysiological conditions among the AA population. TGFβ1 serves as a central molecule for various cellular signaling events including the pathways associated with mTOR and ERK-1/2. Racially disparate expression of TGFβ1 has already been reported and it is highly plausible that TGFβ1 might mediate its effects by modulating mTOR/ERK-1/2 pathways. TGFβ1 promotes epithelial to mesenchymal transition (EMT) through the activation of PI3K/Akt and mTOR signaling cascades, which further enhances distant metastasis (Lamouille and Derynck, 2007; Xu et al., 2009). The activation of the Akt and mTOR-signaling pathways leads to enhanced cancer cell growth (increased protein synthesis), and proliferation (increase in cell number; Lamouille and Derynck, 2007, 2011).

Interestingly, TGFβ is considered a double-edged sword in various human cancers including renal cancer (Akhurst and Derynck, 2001). Initially, TGFβ was reported to be a tumor suppressor, but later its novel oncogenic functions were discovered (Akhurst and Derynck, 2001). So far, there are no direct reports on the racially disparate expression or regulation of mTOR or ERK1/2 pathway. This manuscript is the first report connecting unappreciated essential molecules that are associated with mTOR and ERK-1/2 pathways that are involved in the regulation of various cellular processes including cell survival, differentiation, metabolism, and motility. TGFβ influences major cellular processes in cancer cells including proliferation, survival, epithelial-mesenchymal transition (EMT), and distant metastasis. TGFβ affects the regulation of the ERK-1/2 pathway. Treatment of TGFβ increases the phosphorylation of ERK-1/2, which is reported to further induce EMT in normal murine mammary gland epithelial cells and mouse cortical tubule epithelial cells (Xie et al., 2004).

Several studies have demonstrated TGFβ1 overexpression in AA patients with different disease conditions including renal disease, diabetes, hypertension, and breast cancer compared to CA counterparts (August et al., 2000; Suthanthiran et al., 2000; August et al., 2009; Huan et al., 2010; Quan et al., 2014). Importantly, AA population entails a SNP at codon 10 that is linked to higher levels of both mRNA and protein expression of TGF-β (Suthanthiran et al., 2000; Kim et al., 2004; Stuelten et al., 2005). Furthermore, an increased level of TGFβ1 expression was reported in AA prostate cancer patients compared to the CA counterparts (Elliott et al., 2018). Additionally, increased expression of TGFβ3 was detected in AA-derived prostate cancer cell lines compared to CA-derived cell lines (Elliott et al., 2018). AA prostate cancer patients also exhibited a higher level of TGFβ3 in blood sera compared to the CA cohort (Elliott et al., 2018). TGF-β-1, 2, and 3 are isoforms that interacts with each other and regulate a variety of cellular processes including cell differentiation, migration, invasion, and immune cell functions. However, there is no report on their disparate expression and redundant functions in cancer (De Caestecker et al., 2000; Jung et al., 2017).

Cancer cells often evade immune surveillance and immune clearance through a number of ways, one such approach is the expression of TGF-β. Considering its role in immunosuppression, TGF-β has been developed as a potential therapeutic target in onco-immunotherapy (Ungefroren, 2019). Moreover, TGF-β has been reported to increase the expression of an immune checkpoint molecule, programmed death ligand (PD-L1) in different cancers including lung cancer and pancreatic ductal adenocarcinoma (Hussain et al., 2021; Pan et al., 2021). TGF-β signaling in the tumor microenvironment is associated with immunologically cold tumors and contributes in the development of immune checkpoint resistance (Larson et al., 2020). Preclinical data using mouse models revealed that combination of immune checkpoint blockade with TGFβ inhibition improved the overall anti-tumor response (Ganesh and Massague, 2018). Further, TGF-β could induce immune suppression by promoting T cell-exclusion and Th1 effector functions in various cancers including colon and urothelial cancer (Tauriello et al., 2018; Bai et al., 2019). Taken together, the disparate high expression of TGFβ protein may be used as a personalized biomarker for AA prostate cancer patients for better diagnosis and prognosis of the disease.

Tumor suppressors like PTEN are associated with favorable prognosis and lower risks for cancer development (Maxwell et al., 2000; Charan et al., 2020b). PTEN negatively regulates PI3K and its downstream signaling, mediated through Akt and mTOR. Mutation in PTEN gene abolishes its protective functions and is often reported in various human cancers. The frequency of PTEN mutations in endometrial cancer patients was significantly higher in CA population than AA cohort (Maxwell et al., 2000). In addition, the rate of PTEN loss in prostate cancer patients was significantly higher in European American (EA) men than AA counterpart (Tosoian et al., 2017). KRAS is a family member of Ras genes. RAS proteins are GTPases that regulate survival, cell cycle, migration, cytoskeletal reorganization, and vesicular transport. KRAS is activated by epidermal growth factor receptor (EGFR) that further activates MEK/ERK1/2 and mTOR pathway (Tomasini et al., 2016). KRAS mutations in lung cancers are more common in AA patients than CA patients (Staudacher et al., 2017) that make mTOR a potential therapeutic target for KRAS-mutant lung cancer.

Recently, the differential expression of matrix metalloproteinases (MMP) family proteins, especially MMP2 and 9 has been studied in the different ethnic groups of cancer patients. Interestingly, the role of mTOR and ERK-1/2 signaling in regulating the expression of MMPs has been reported in different solid tumors (Chen et al., 2009; Akter et al., 2015; Xia et al., 2015; Park et al., 2016; Tsao et al., 2018). Besides, TGFβ that is a key mediator of mTOR/ERK-1/2 associated signaling cascades, has been shown to regulate MMP2 and MMP9 expression (Moore-Smith et al., 2017). The function of MMP2 and MMP9 are very well-characterized in cancer cell metastasis. Racially disparate expression of MMP2 and 9 has been reported in AA prostate cancer patients (Elliott et al., 2018; Ganaie et al., 2018). In addition, AA-derived prostate cancer cell lines showed higher expression of MMP2 and MMP9 both at transcriptome and proteome levels compared to CA counterparts (Elliott et al., 2018).

Several chemical inhibitors against different MMPs including MMP2 and 9 have been developed as potential antitumor agents (Tsao et al., 2018). mTOR and ERK-1/2 signaling pathways regulate metastasis of cancer cells by modulating MMPs (Park et al., 2016; Zou et al., 2020). *Salmonella* treatment inhibited AKT/mTOR signaling pathways, which further reduced the expression of MMP9 in the mouse tumor models (Tsao et al., 2018). Interestingly, the treatment of Chrysin (ERK-1/2 and JNK inhibitor) also reduced the expression of MMP9 in gastric cancer cells (Xia et al., 2015). Altogether, TGFβ and MMPs may act as key players in influencing mTOR and ERK-1/2 functions in cancer cells and their disparate expressions may predict different clinical outcomes.

AA-prostate cancer patients have also been shown to express higher levels of EGFR compared to CA prostate cancer patients (Shuch et al., 2004). EGFR is an upstream regulator of mTOR and ERK-1/2 signaling cascade and its overexpression and aberrant mutational status have been reported in several human cancers including renal cancer (Cossu-Rocca et al., 2016; Sooro et al., 2018). Furthermore, mTOR inhibitors reduced the proliferating potential of EGFR mutant drug-resistant lung cancer cells (Ishikawa et al., 2013). Moreover, ERK-1/2 signaling chemo-sensitizes drug-resistant lung cancer cells to WZ4002 (EGFR inhibitor). In addition, ERK-1/2 signaling attenuates the emergence of drug resistance in lung cancer cells. Another important regulator of the mTOR and ERK-1/2 signaling pathway is AKT, which is commonly altered among the CA cohort of breast cancer patients compared to AA counterparts (Khan et al., 2013). AKT promotes Ras/Raf/ERK-1/2 and mTOR signaling cascades, which in turn enhances the proliferation and survival of cancer cells (Chang et al., 2019).

Cross-talk between MAPK signaling pathways with other signaling pathways significantly impacts the clinical outcome of targeted anti-cancer therapies. ERK1/2 shares a number of proteins that interact with MST kinase, which is a key protein molecule of the Hippo signaling pathway. The Hippo signaling pathway primarily plays an important role in development. Dysregulation of this pathway can leads to the formation of tumor (Han, 2019). ERK1/2 and MST commonly mediate their effects through MYC, FOXO3, and TP53 proteins for regulating metabolism, growth, and cell death (Harvey et al., 2013). Higher genetic variations in the Hippo signaling pathway were reported in AA women than CA counterpart (Zhang et al., 2016). Hippo pathway alterations may also contributes in elevating breast cancer risks in AA women. In addition, Son of Sevenless 1 (SOS1) is an activator of Ras/MAPK, which is overexpressed in AA prostate cancer cells. SOS1 increased levels caused higher cancer cell proliferation and migration through increased activation of ERK signaling pathways (Timofeeva et al., 2009).

PI3K-AKT is one of the most important signaling pathways, which is differentially activated among different races and may modulate the mTOR pathway in several human cancers. AA renal cancer patients, especially, clear cell type-B (ccB), demonstrate higher activation of von Hippel-Lindau (VHL) and reduced expression of hypoxia-inducible factor (HIF) gene signature compared to CA patients (Krishnan et al., 2016). Such AA clear cell renal cancer patients show minimal response to VEGF-directed therapy as compared to CA counterparts. This could be one of the important reasons that contribute to the worst survival rate of AA patients receiving anti-VEGF therapy for clear cell renal carcinoma. In addition to VHL, mTOR and PI3KCA genes were altered in tumor samples of clear cell renal cell carcinoma (ccRCC) patients. The upregulation of the HIF pathway also leads to activation of tyrosine receptor kinases (RTKs), which initiates the signaling cascade of the Raf/MEK/ERK-1/2 pathway in renal carcinoma cells (Oka et al., 1995). In conclusion, the cross talk between HIF and mTOR/ERK-1/2 signaling pathways may regulate the aggressive properties of renal carcinoma in the AA population. In addition, higher activation of ERK in colorectal cancer and melanoma indicates poor responses to both neoadjuvant and adjuvant chemotherapy (Mirmohammadsadegh et al., 2007; Holck et al., 2019). Hence, ERK1/2 inhibition in combination with chemotherapy may turn out more effective anti-tumor therapeutic strategy. Overall, disparate expression of mTOR and ERK-1/2 associated signaling proteins can be selectively targeted along with mTOR or ERK-1/2 inhibitors to efficiently inhibit the tumor growth, metastasis, and to increase the overall survival depending upon the specific race.

## Conclusion

In this review article, we attempted to shed light on disparate expression of unrecognized essential signaling molecules related to mTOR and ERK-1/2 pathway that play an important role in regulation of various cellular processes. AA groups of patients are at higher risk of developing highly invasive and aggressive cancers than CA cancer patients. mTOR and ERK-1/2 pathways have been the most extensively studied pathways in different cancers and their targeting has given promising results. mTOR and ERK-1/2 regulate cancer cells aggressiveness through modulating the multiple signaling pathways in human body. Furthermore, the differential expression of mTOR and ERK-1/2 associated signaling molecules may significantly affect the clinical outcome of certain AA cancer patients and could be used in precision medicine to improve the therapeutic response and overall survival. Moreover, this information will help in designing innovative treatment strategies against various aggressive cancers to improve the clinical outcome for different racial groups. Overall, this comprehensive study will unleash unappreciated regulators or pathways involved in various pathophysiological processes and could lead to the development of effective targeted therapy aimed at improving the quality of life of cancer patients from different ethnicity.

## Author Contributions

All authors contributed to writing and drafting the manuscript. SM, MC, BR, AV, DA, and RG conceived and reviewed the final version of the manuscript. All authors contributed to the article and approved the submitted version.

### Conflict of Interest

The authors declare that the research was conducted in the absence of any commercial or financial relationships that could be construed as a potential conflict of interest.

## Abbreviations

AA: African American
CA: Caucasian American
SNP: Single-nucleotide polymorphism
mTOR: Mechanistic target of rapamycin
ERK-1/2: Extracellular-signal-regulated kinase
TGF-β: Transforming growth factor-β
EMT: Epithelial to mesenchymal transition
EGFR: Epidermal growth factor receptor
MMP: Matrix metallo proteinase
MAPK: Mitogen activated protein kinase
